# Xenoimplantation of an Extracellular-Matrix-Derived, Biphasic, Cell-Scaffold Construct for Repairing a Large Femoral-Head High-Load-Bearing Osteochondral Defect in a Canine Model

**DOI:** 10.1155/2014/127084

**Published:** 2014-03-11

**Authors:** Yang Qiang, Zhao Yanhong, Peng Jiang, Lu Shibi, Guo Quanyi, Ma Xinlong, Xia Qun, Xu Baoshan, Zhao Bin, Wang Aiyuan, Zhang Li, Xu Wengjing, Zeng Chao

**Affiliations:** ^1^Institute of Orthopedics, Chinese PLA General Hospital, Beijing 100853, China; ^2^Department of Spine Surgery, Tianjin Hospital, Tianjin 300211, China; ^3^Stomatological Hospital of Tianjin Medical University, Tianjin 300070, China

## Abstract

This study was aimed to develop an ECM-derived biphasic scaffold and to investigate its regeneration potential loaded with BM-MSCs in repair of large, high-load-bearing osteochondral defects of the canine femoral head. The scaffolds were fabricated using cartilage and bone ECM as a cartilage and bone layer, respectively. Osteochondral constructs were fabricated using induced BM-MSCs and the scaffold. Osteochondral defects (11 mm diameter × 10 mm depth) were created on femoral heads of canine and treated with the constructs. The repaired tissue was evaluated for gross morphology, radiography, histological, biomechanics at 3 and 6 months after implantation. Radiography revealed that femoral heads slightly collapsed at 3 months and severely collapsed at 6 months. Histology revealed that some defects in femoral heads were repaired, but with fibrous tissue or fibrocartilage, and femoral heads with different degrees of collapse. The bone volume fraction was lower for subchondral bone than normal femoral bone at 3 and 6 months. Rigidity was lower in repaired subchondral bone than normal femoral bone at 6 months. The ECM-derived, biphasic scaffold combined with induced BM-MSCs did not successfully repair large, high-load-bearing osteochondral defects of the canine femoral head. However, the experience can help improve the technique of scaffold fabrication and vascularization.

## 1. Introduction

Avascular necrosis of the femoral head (ANFH) is a generally refractory disease of clinical orthopedics and is common in middle-aged people. The disease causes hip pain, eventuallyleading to femoral head collapse and osteoarthritis, ultimately requiring total hip arthroplasty. The causes of ANFH are approximately divided into 2 categories: traumatic osteonecrosis, due to a sudden interruption of blood supply to the femoral head, and nontraumatic ANFH, with progressive and chronic process. In addition, hormone drugs can also lead to the disease [1]. The common feature of these diseases is defective blood supply to the femoral head.

Despite the multiple methods of treating ANFH, a number of cases are not successfully treated, so femoral heads continue to collapse, thus leading to loss of function of hips [2–6]. In addition, osteochondral femoral joint damage is common, but traditional surgical methods of repair are ineffective. With tissue engineering as well as biomechanical research, treatment of ANFH and femoral-head cartilage damage may be possible [7, 8]. The core premise of tissue engineering is the use of a small number of cells greatly amplified* in vitro* combined with biological materials to construct bioactive bone tissue to repair defects [9].

**Figure 1:**
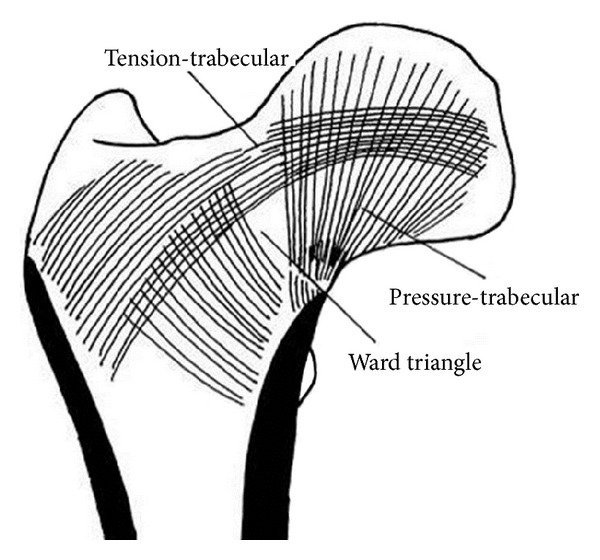
Model of the femur with orientation structure based on biomechanical research.

**Figure 2:**
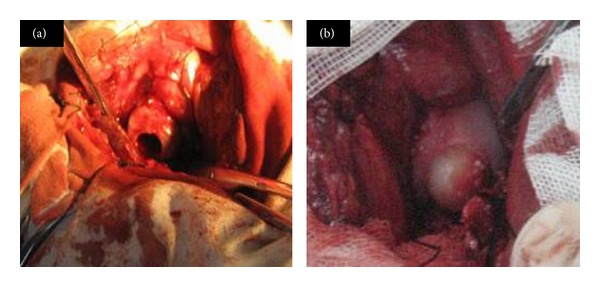
Implantation of the cell-scaffold complexities: (a) preparation of osteochondral defects in high-load-bearing area of canine femoral head and (b) osteochondral implantation.

In this study, we aimed to (1) prepare and characterize an extracellular-matrix-(ECM-)derived porous and integrated biphasic osteochondral scaffold with the orientation structure of natural femoral trabecular bone (Figure 1) and (2) investigate the regenerative ability of such a scaffold loaded with chondrogenically induced bone-marrow mesenchymal stem cells (BM-MSCs) to repair large, high-load-bearing osteochondral defects of the femoral head in a canine model. In combining bone tissue-engineered cartilage with BM-MSCs, we could evaluate the use of such complexes to repair high-load-bearing osteochondral defects in the large femoral heads of canine models.

## 2. Materials and Methods

### 2.1. Fabrication of Structural Bone-Cartilage Biphasic Scaffolds

Suspensions of microfilaments of decellularized cartilage matrix (DCM) were prepared as we previously described [10]. Soaked decellularized cancellous bone matrix (DCBM) columns were placed into cylindrical silicon molds with a 3% (w/v) DCM microfilament suspension to generate a biphasic structure. The molds were frozen at −20°C and −80°C for 1 h each and then lyophilized (FD-1, Boyikang; Beijing, China) for 48 h. The scaffolds were cross-linked by dehydrothermal treatment and with a carbodiimide solution (EDC). ECM-derived DCM/DCBM biphasic scaffolds were 11 mm diameter and 10 mm depth and were sterilized with ^60^Co *γ* irradiation.

### 2.2. Characterization of ECM-Derived Biphasic Scaffold Microstructure

To observe the microstructure of the ECM-derived DCM/DCBM biphasic scaffold, specimens were cut from the scaffolds and the interior microstructure of cross-sections was investigated by scanning electron microscopy (SEM, Hitachi S-520, Japan) after coating with gold-palladium. Porosity was measured by the ethanol intrusion method. To observe the inner microstructure of the scaffold, we used micro-CT (GE Medical Systems, London, ON, Canada) [11]. Specimens were immersed in a 3% OsO4 solution to increase the X-ray attenuation and then air-dried.

### 2.3. *In Vitro* Cell Culture Studies

#### 2.3.1. Chondrogenic Induction of BM-MSCs

BM-MSCs were obtained from dogs (2 years old, provided by the Institutional Review Board of the Chinese PLA General Hospital) and isolated as described [10]. BM-MSCs (passage 3) were cultured and induced with conditioned medium (10 ng/mL fibroblast growth factor, 10 ng/mL transforming growth factor *β*1, 50 mg/L vitamin C, 10^−7 ^mol/L dexamethasone, 1% insulin-transferrin iron selenium, and 10% fetal bovine serum, all from Sigma). The biphasic scaffolds were placed into 6-well plates and induced for 2 weeks.

#### 2.3.2. Cultivation of Cell-Scaffold Complexes

With 2 weeks of induced culture, the cartilage-like layer of scaffolds was seeded with induced cells at 5 × 10^7^/mL after rinsing with chondrogenic medium for 20 min. The cell-scaffold complexes were incubated for 1 h at 37°C in 5% CO_2_, and 5 *μ*L medium was added every 30 min to prevent cell death before adding culture medium. The medium was replaced twice a week.

#### 2.3.3. Cell Attachment to the Scaffolds

The cell-scaffold constructs were fixed with 2.5% glutaraldehyde and then osmic acid, dehydrated in a graded ethanol series and dried by critical-point drying, and then sputter-coated with gold-palladium before observation. Cell attachment was observed by SEM (X-650, Hitachi, Japan).

#### 2.3.4. Cell Viability

A cell survival kit (Molecular Probes; Eugene, OR) was used for examining cell viability in scaffolds. After 72 h of culture, cell-scaffold complexes, 100 *μ*m, thick were incubated with fluorescent solution (2 mM ethidium Homodimer-1 and 4 mM calcein AM). Samples were observed under a Leica confocal microscope (Wetzlar). Viability of cells was determined by proportion of viable cells (green cells) and nonviable cells.

### 2.4. *In Vivo* Studies

#### 2.4.1. Preparation of Canine Model and Implantation of Scaffolds

We used 12 male canines (20–25 kg). The study was approved by the Institutional Animal Care and Use Committee of the Laboratory Animal Research Centre, Chinese PLA General Hospital. Canines received Sumianxin II (0.08–0.10 mL/kg) and ketamine (40 mg/kg) via intramuscular injection. An anterolateral incision of the skin, muscle, and joint capsule of the hip revealed the femoral head; we prepared a joint osteochondral defect (diameter 11 mm, depth 10 mm) (Figure 2(a)) in the main high-load-bearing area of the femoral head (between the round ligament and outer edge of the femoral head) with use of a sharp trephine (diameter 11 mm) and implanted cell-scaffolds into the defect (Figure 2(b)). The contralateral hip was the normal control. Dogs were given an intramuscular injection of penicillin (1.6 million U) once a day for one week and allowed free movement. Animals were observed for overall activity, and range of motion was compared between normal and implanted hips.

#### 2.4.2. Radiography and Gross Morphological Observation

After 3 and 6 months, animals (6 dogs at each time) were anesthetized intramuscularly with Sumianxin II, 2 mL/Kg. We observed the repair of the hip anteroposterior region by radiography. All radiographs involved the same batch of negatives and the same exposure conditions. Radiography was followed by general morphological observation. Dogs were given tetracycline solution (30 mg/Kg) via muscular injection at 14 days; then 3 days later, they were anesthetized and killed. Blood samples were removed from the femoral artery and material was extracted from the bilateral femoral head. After general observation, the femoral head was excised along the longitudinal axis of the femoral neck to observe the repair of the femoral bone defect.

#### 2.4.3. Histology

Specimens at 3 and 6 months were fixed with 10% formalin for 24 h, dehydrated with a graded ethanol series, then embedded in paraffin and sectioned in 10 mm thick slices, and stained with hematoxylin and eosin (HE) and toluidine blue (Sigma).

#### 2.4.4. Micro-CT Scanning

We used 4 samples for each time (3 and 6 months), with the normal femoral head (*n* = 6) as a control. Samples were placed in the test tube of the micro-CT system (American GE) and scanned at resolution 45 *μ*m along the longitudinal direction for successive micro-CT images. Cylindrical regions of interest (ROI) of 11 mm with height 10 mm coincided with the surgical implantation site; then we performed 3D reconstruction, with analysis by a microview package. ROI bone volume fraction (BVF) of subchondral bone was calculated as bone volume (BV)/sample volume (TV).

#### 2.4.5. Assessment of Biomechanical Properties for Repairing Femoral Head Tissue

We used 4 samples (6-month group) and 6 samples (normal canine femoral head) for examination. We used an MTX machine for measurement and ensured that the force lines of the loading device were perpendicular to the cartilage surface (Figure 10); maximum pressure was 60 N and speed of pressure 0.01 mm/s. Before each loading, samples were preloaded with 10 N force 3 times to obtain a mechanical curve. Bone stiffness was calculated as the ratio of loading force and bone deformation (*K* = Δ*F*/Δ*T*), calculated by the load-displacement curve. We selected 3 different points of the same femoral head to calculate the mean.

### 2.5. Statistical Analysis

Data were analyzed by SPSS 11.0 (SPSS Inc., Chicago, IL, USA) and expressed as mean ± SD. *P* < 0.05 was considered statistically significant.

## 3. Results

### 3.1. Characterization of ECM-Derived Biphasic Scaffold

**Figure 3:**
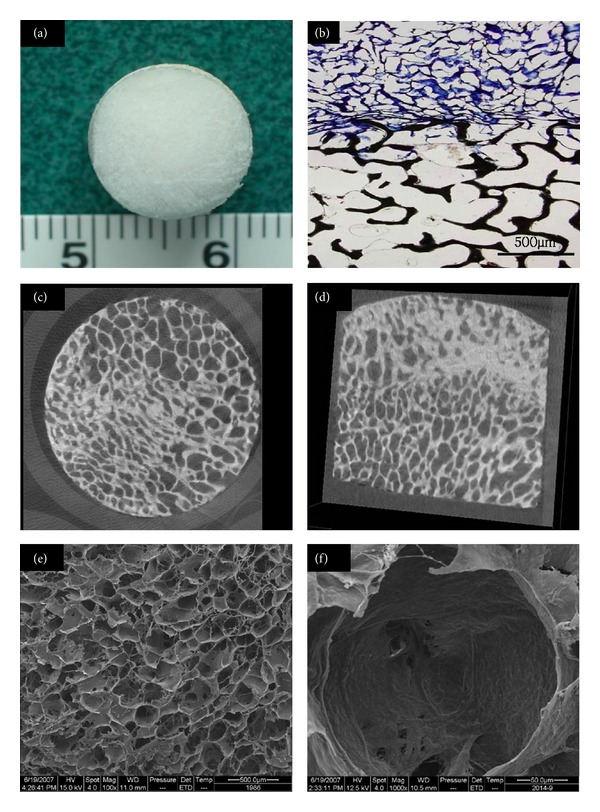
Scaffold structure and histology: (a) morphology of the scaffold, (b) Giemsa staining of nondecalcified section (×100), (c) cross-section of micro-CT image, (d) longitudinal section of micro-CT image, and scanning electron microscopy (SEM) of cartilage layer of scaffold (e) (×100) and (f) (×1000).

We observed the general shape of the biphasic scaffold (Figure 3(a)). Histological results revealed a uniform pore with good connection between the cartilage and bone layer (Figure 3(b)). The cartilage layer of the scaffolds formed a typical spongy 3D porous interconnected structure that retained most of the ECM without cells or cell debris (Figure 3(b)). A uniform pore with good connection was observed in the bone layer from the micro-CT results (Figures 3(c) and 3(d)). SEM also revealed uniform porosity in cartilage layer (Figures 3(e) and 3(f)). The average pore size in the porous cartilage layer was 231.6 ± 57.2 *μ*m and average porosity 89.3 ± 2.0%.

### 3.2. Cell Attachment on Scaffolds

**Figure 4:**
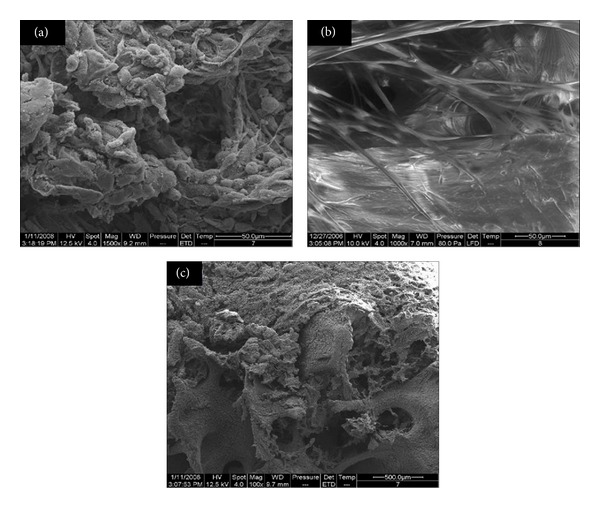
SEM of cell attachment on scaffolds: cell adhesion of (a) cartilage layer (×1500), (b) bone layer (×1000), and (c) both layers (×100).

After 72 h of culture, SEM revealed chondrogenic BM-MSCs attached on the biphasic scaffold. There were many round or elliptical cells that resembled chondrocyte-like cells, and cells secreted a large number of matrices both on the cartilage and bone layers (Figures 4(a), 4(b), and 4(c)).

### 3.3. Cell Viability in Porous Scaffold

**Figure 5:**
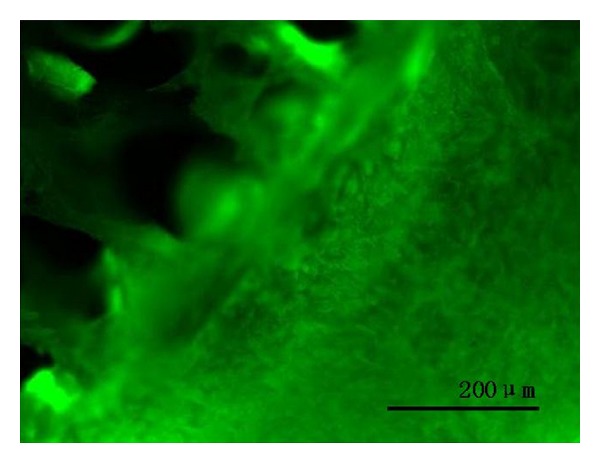
Confocal laser scanning microscopy of cell-scaffold complexes at 72 h after seeding. Complexes were incubated with fluorescent solution (2 mM ethidium Homodimer-1 and 4 mM calcein AM). Green indicates living cells. Bar = 200 um.

Live/dead staining revealed cell activity on the biphasic scaffolds after 72 h* in vitro* culture, with no dead cells (Figure 5).

### 3.4. *In Vivo *Animal Study

No dogs died during the experiment, and wounds healed well. At 3 months after surgery, hip range of motion on the surgical side was not significantly limited. Dogs showed a slight limp when walking. After 6 months, dogs showed significantly limited range of motion on the surgical side and obvious limping.

### 3.5. Radiography and Gross Morphological Observation

**Figure 6:**
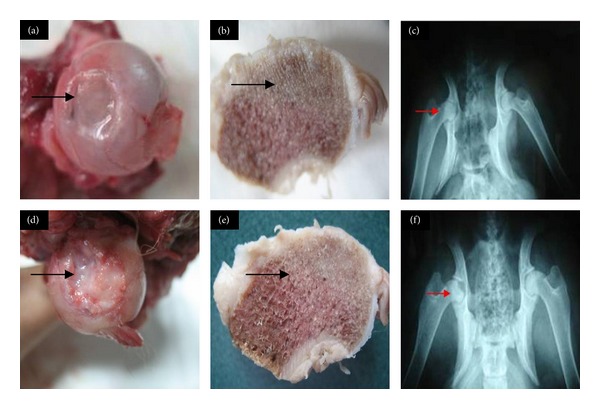
Radiography and gross morphology of canine femoral heads: gross morphology of cartilage at (a) 3 months after repair and (b) bone at 3 months; (c) radiography of the femoral head at 3 months; gross morphology of cartilage at (d) 6 months after repair and (e) bone at 6 months; (f) radiography of the femoral head at 6 months.

At 3 months, the whole high-load-bearing area of the femoral head showed mild collapse on radiography and gross morphological observation (Figures 6(a), 6(b), and 6(c)). The shape of the femoral head was intact and the repair area was depressed and not smooth. Mild osteoarthritis was visible in other parts of the femoral head. The repair of bone indicated insufficient blood supply. At 6 months, severe collapse could be seen on radiography and gross morphology. The shape of the femoral head was lost and the femoral head showed severe osteoarthritis changes (Figures 6(d), 6(e), and 6(f)). Blood supply to the bone layer was insufficient. The entire collapse of the femoral head and a large formation of osteophytes eventually led to severe cartilage damage.

### 3.6. Histology

**Figure 7:**
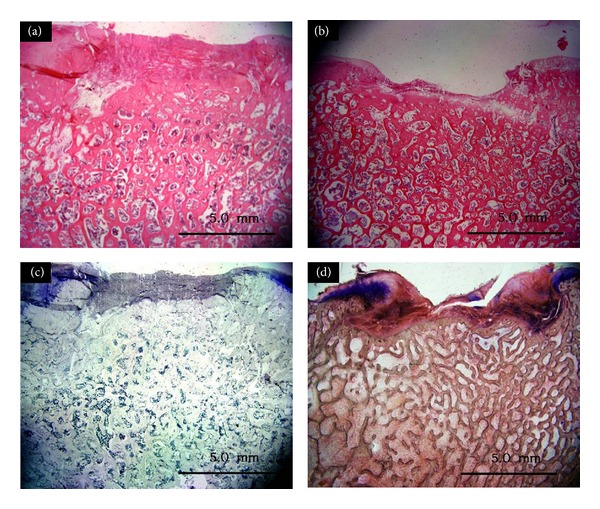
Histology of cell-scaffold complexes: hematoxylin and eosin staining at (a) 3 months and (b) 6 months; toluidine blue staining at (c) 3 months and (d) 6 months. Bar = 5 mm.

Toluidine blue and HE staining at 3 months showed some cartilage-defect sections filled with fibrous tissue and some fibrocartilage; the remaining tissue was fibrocartilage or fibrous tissue. The surface was uneven and ECM was not stained, with disintegration or cystic degeneration. New tissue was poorly combined with surrounding cartilage containing no cells, for degenerative changes of cartilage cell proliferation. Mild collapse was found in the bone-defect sections and an internal trabecular bone structure existed but without the normal orientation of the femoral head (Figures 7(a) and 7(c)). At 6 months, some cartilage-defect sections were filled with fibrous tissue. The surface was uneven and ECM was not stained, with disintegration or cystic degeneration. A crack defined the surrounding tissue and cartilage cell proliferation was obvious. Collapse could be seen in the bone-defect sections. Trabeculation existed and scaffolds stood tightly with the surrounding bone with part of the trabecular stents absorbed (Figures 7(b) and 7(d)).

### 3.7. Tetracycline Fluorescence Observation of Nondecalcified Sections

**Figure 8:**
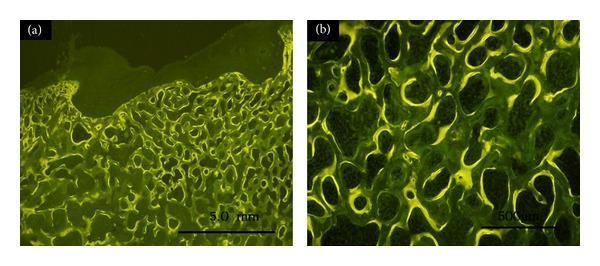
Tetracycline fluorescence labeling of new bone under fluorescence microscopy at 6 months: (a) (×12.5), (b) (×100).

Tetracycline fluorescence labeling revealed new bone formation in the repair area at 6 months, tightly combined with the surrounding host bone, although with more obvious collapse at 6 months (Figure 8).

### 3.8. Micro-CT Analysis

**Figure 9:**
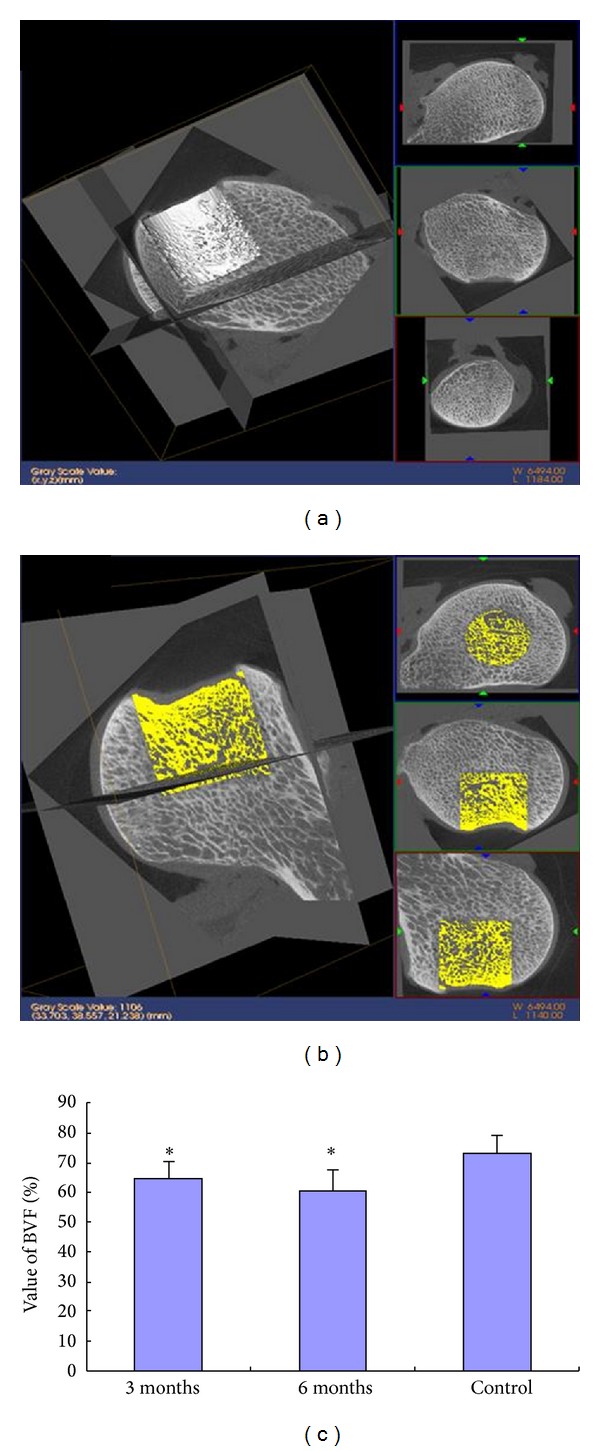
Micro-CT analysis: micro-CT images of implantation in (a) defective femoral head at 3 months and (b) defective femoral head at 6 months; (c) percentage of bone volume fraction (BVF) (%) at 3 and 6 months after repair of femoral head. *P < 0.05 compared with the control.

Micro-CT reconstruction revealed collapse of high-load-bearing areas both at 3 and 6 months (Figures 9(a) and 9(b)). BVF value at different times were lower in treated than in normal femoral heads (*P* = 0.0430 and *P* = 0.0116 at 3 and 6 months, resp.).

### 3.9. Assessment of Biomechanical Properties for Repairing Femoral Head Tissue

**Figure 10:**
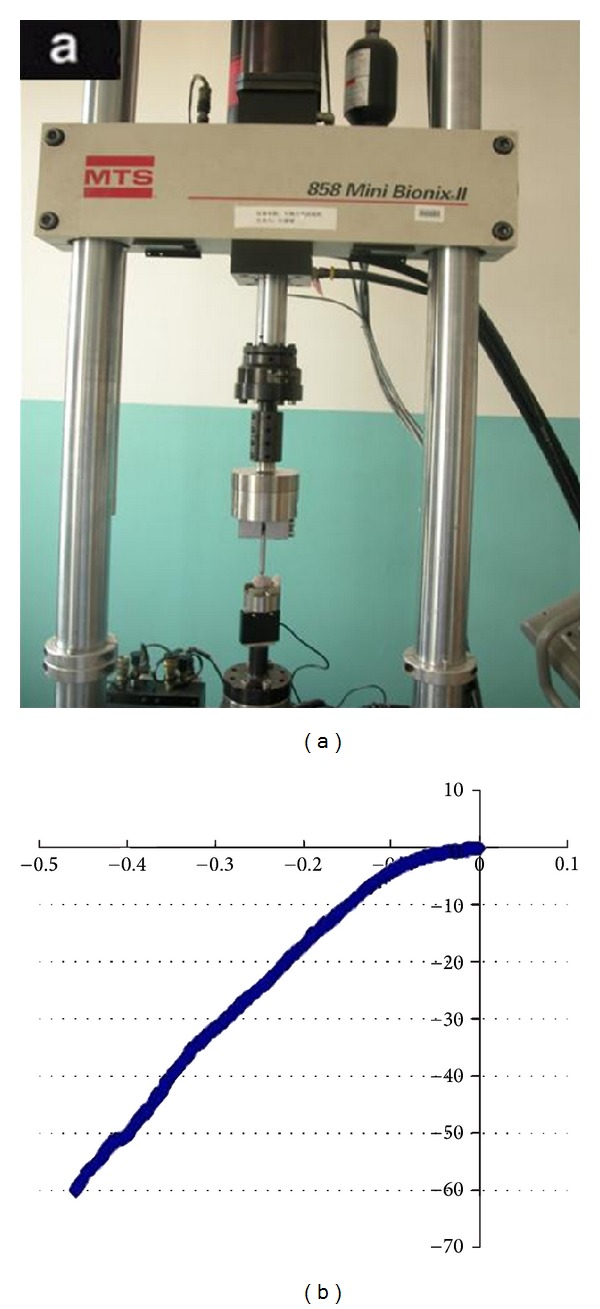
Biomechanical properties test: (a) device for mechanical testing, (b) load-displacement curve.

On the basis of the load-displacement curve (Figure 10), the stiffness of normal high-load-bearing area was higher for normal femoral head bone tissue than 6-month high-load-bearing subchondral bone tissue (235.13 ± 35.56 versus 134.76 ± 43.12 N/mm, Table 1), only 57.3% of the contralateral normal femoral head (*P* = 0.0038).

## 4. Discussion

We developed an ECM-derived, integrated biphasic scaffold using ECM cartilage as a cartilage layer and acellular bone matrix as a bone layer. The biphasic scaffold was seeded with bone-marrow mesenchymal stem cells (BM-MSCs) induced for 72 h. Osteochondral defects on articular high-load-bearing femoral heads from dogs were filled with the cell-scaffold constructs. Cells grew well on the scaffold. Histology revealed that some femoral heads with cartilage defects were repaired, but with fibrous tissue or fibrocartilage and severe osteoarthritis, and femoral heads with different degrees of collapse. The bone volume fraction was lower for subchondral bone than normal femoral heads at 3 and 6 months, and rigidity was lower in repaired subchondral bone than normal femoral heads at 6 months. The ECM-derived, integrated biphasic scaffold combined with chondrogenically induced BM-MSCs did not successfully repair large, high-load-bearing osteochondral defects of the femoral head, but the experience can help improve the technique of scaffold fabrication and vascularization.

Early treatment of ANFH is difficult. Currently, many surgical methods to protect the hip include core decompression [12], vascularized implantation [13], and resurfacing [14]. Many traditional surgical repair methods have been ineffective and are more for early treatment and not associated with the collapse of the necrotic head. For the patients of ARCO III period (“crescent sign,” femoral head collapse, not accompanied by osteoarthritis). The choice of surgical approach is controversial. With the development of molecular biology, stem cell technology and materials science, and other related disciplines, tissue engineering technology offered great hope for the treatment of ANFH and large areas of osteochondral defects. The principle is the preparation of bone tissue-engineered cartilage cells combined with the seed and the use of composite implants to repair necrosis or osteochondral defects in the defective site. The principle is orthotopic transplantation to repair defects.

The microstructure of trabecular bone of the high-load-bearing area is important in repair [8, 15, 16]. In accordance with the principle of bionics, the mechanical properties and structure of the bone graft used to repair femoral head necrosis or osteochondral defects should be similar to normal high-load-bearing areas. In this study, the elastic modulus, strength, and other mechanical properties of the bone column from the allogeneic canine femoral head were similar to fresh bone. In previous study, we used an acellular osteochondral scaffold to successfully repair osteochondral defects of the canine knee [17]. The 3D structure of the scaffold was conducive to growth of new capillaries and bone cells [18].

In this study, we prepared natural, decellularized, ECM-derived DCM/DCBM biphasic biomaterial as a scaffold for bone repair. This biphasic scaffold had an interconnected and porous microstructure that was similar to the intrinsic structure of the ECM of osteochondral tissue. More prominent was the stability at the interface of the cartilage-bone layers and part of the impregnated DCM and DCBM that determined its overall integrity.

We selected BM-MSCs as the seed cells because BM-MSCs have strong self-proliferation and differentiation potential and they can differentiate into bone, cartilage, fat and muscle cells, and other cell lineages [19–21]. Adhesion and proliferation of cells on the biphasic scaffolds was a basement for the repair and reconstruction of bone tissue. Culture in the 3D porous scaffolds is affected by physical and chemical properties of materials, surface microstructure, and the microenvironment cells are cultured in. SEM revealed that cells attached on the scaffold with good growth and proliferation and secreted a large amount of ECM, so the ECM-derived acellular biphasic scaffold provided a good environment for the growth of induced BM-MSCs. Live/dead staining was used to observe the activity of cell lactonase and integrity of the cell membrane. The activity of induced BM-MSCs in biphasic scaffolds was detected, with no dead cells.

We used a tissue engineering approach to reconstruct bone tissue with physiological function in defective canine femoral heads. Cui et al. [22] amplified BM-MSCs* in vitro* and then implanted them in autologous bone defects in a rat model, achieving good results. Tampieri et al. [23] prepared a biomimetic osteochondral composite scaffold consisting of a lower layer of biomineralized collagen and an upper layer of hyaluronic acid-charged collagen that mimicked the cartilaginous region. Rijnen et al. [24] filled subchondral bone defects in femoral heads of 15 goats with cancellous bone and CPC and found that it could prevent early collapse of femoral head. Maddox et al. [25] prepared allograft DBM as a scaffold, combined with human cultured MSCs, which was implanted subcutaneously into nude mice to detect the features of ectopic bone.

Canine has been used in research of various treatment methods to protect the femoral head because of the size of dogs, diameter of femoral head, and the high-load-bearing femoral head, which is relatively large. We selected acellular bone with a trabecular bone structure as the scaffold and ECM as the cartilage to prepare an osteochondral biphasic scaffold seeded with BM-MSCs for repairing ANFH and a large area of osteochondral defects. Gross observation, radiography, tetracycline fluorescence assay, histology, micro-CT, and biomechanical testing revealed that the repair with the scaffold was not satisfactory at 3 or 6 months after repair. Osteoarthritis changes could be seen on the articular surface of the femoral head and the high-load-bearing area, and the entire femoral head collapsed. The pathological change of articular cartilage resulted from the collapse as well as damage of the bone structure [1]. Despite the internal trabecular bone structure, the repaired femoral head had no structure of the normal femoral head and showed significantly decreased biomechanical strength.

We analyzed the reasons for the failure. First, although we used acellular bone, which was similar to the structure and mechanical properties of an allogeneic femoral-head weighting-bearing area for remodeling, it features sustained and repeated loads on the bone. Allogeneic bone* in vivo* degrades too fast for timely repair. We found multiple fatigue fractures, osteonecrosis, and collapse of femoral head. The subchondral bone must have mechanical support during the repair of femoral-head necrosis. Second, the blood supply to the femoral head was relatively poor and vulnerable, which resulted in avascular necrosis. In* in vitro* culture of cell-scaffold complexities, ischemic necrosis often occurred in the core parts. The survival of tissue-engineered bone implants to repair large bone defects depends on blood flow and interstitial fluid infiltration, and infiltration of tissue fluid should be <200 *μ*m [26]. The function of seed cells may be impaired or cells may be dead due to lack of nutrition, so implantation was with pure bone-conduction material for difficultly in achieving the desired repair effect. In this study, the regeneration of a large femoral-head high-load-bearing osteochondral defect in a canine model was insufficient, and nutrients could not reach the middle of the bone column, which contributed to the collapse. Surgery to prevent collapse of the femoral head with avascular necrosis is a hot topic and difficult to study [27]. With apparent collapse of the femoral head, resurfacing was a method to replace cartilage by prosthesis; retaining most of the femoral head and femoral neck was considered to be a bridge to replacement of the total hip [28]. In our study, restricting the weight-bearing activities of the femoral head in dogs after surgery was difficult, which may have led to collapse of the femoral head and osteoarthritis.

## 5. Conclusions

In summary, we used tissue engineering to reconstruct osteochondral defects in femoral heads of dogs as preliminary experiments and exploratory research. Although the final restoration was poor, analyzing the reasons for failure helped further improve our scaffolds. A bionic scaffold with suitable mechanical strength and good degradation rate according to the trabecular microstructure of high-load-bearing area combined with vascular technology will improve the effectiveness of restoration and provide a new choice for ANFH and large osteochondral defects of the femoral head.

## Figures and Tables

**Table 1:** Stiffness of bony part in the osteochondral restoration area.

Group	Stiffness (N/mm)
6-week repair (*n* = 4)	134.76 ± 43.12
Normal femoral head (*n* = 6)	235.13 ± 35.56

*P < 0.05 versus control compared to normal femoral head.
